# Hospital Meetings

**Published:** 1896-06-13

**Authors:** 


### HOSPITAL MEETINGS.

### NORTH LONDON HOSPITAL FOR CONSUMPTION.

A festival dinner in aid of the funds of the North London Consumption Hospital was held at the Hotel Cecil on the 9th inst., under the chairmanship of Lord Kinnaird. Nearly 200 guests sat down to dinner. The Chairman, in proposing prosperity to the hospital, said that people, when it was a question of putting the defences of the country on a proper footing, willingly gave millions for that object. How much more should they give liberally when they were face to face with another and more deadly enemy even than war, namely, disease. They were hampered in such a fight, however, by the fact that their enemy could only be attacked in sections. During the last century one section—small-pox—had been successfully attacked, for whereas years ago small-pox was responsible for about 10 per cent. of the mortality of this country, now only about one in 52,000 persons succumbed to that disease. If one section could be so successfully attacked, they might hope in time to gain a decisive victory over another section—consumption—which was responsible for about 13 per cent. of deaths in the present day. The North London Consumption Hospital, a portion of their attacking army, had during the year 1895 treated 434 in and 3,265 out patients at a cost of about £6,000. The income from annual subscribers only amounted to about £1,600, while the Hospital Sunday and Saturday Funds contributed a further £700. This left about £3,700 to be given in donations and other ways, and he hoped that that gathering would bring in the greater part of that sum to the funds of the hospital. (Applause.) The Secretary announced that collections amounting to £2,661 had been received as the result of the dinner.

### PADDINGTON GREEN CHILDREN'S HOSPITAL.

The thirteenth annual meeting of this hospital was held in the board-room on Tuesday, 9th inst. Mr. George Hanbury, the chairman of the hospital, presided, supported by Sir William Hart-Dyke, M.P., and amongst those present were the Revs. C. J. Ridgeway, A. Scott, and C. J. Martin; Messrs. Seton-Karr, George Chance, Balfour Allen, B. A. Willcox, Lionel Hanbury, F. D. Mocatta, Douglas Owen, and Dr. Ogilvie. The Chairman, in moving the adoption of the report, mentioned the fact that this was the second annual meeting that had been held in the present hospital, which, in his opinion, was almost perfect, as it certainly ought to be in view of the great amount of trouble and care that had been taken with the erection of these new buildings. He also pointed out that the hospital was at present free from debt, a wonderful thing, in his opinion, to be able to say of a hospital opened as this had been done. It, however, needed an operation theatre, and to meet this need a new wing was to be added, containing an isolation ward for six beds, an operation theatre, and other accommodation. The cost of this new wing was estimated at about £3,500, and he (the chairman) hoped and believed that this amount might be forthcoming, as also increased income to enable the additional expenses entailed by extra hospital accommodation to be met. Sir William Hart-Dyke, M.P., in seconding the motion, expressed the pleasure which he had felt on going over the hospital, and his feeling that of all the ambitions which animated the various classes of humanity there was no ambition which should more demand constant sympathy and support than that of the man or woman who was constantly sending the hat round on behalf of the institution with which his or her life was happily identified. He referred to the steady growth of the hospital since its foundation in 1862, and to the continuous efforts on its behalf which had resulted in the splendid existing hospital. But it was in the nature of all hospitals to be ever dissatisfied, good and excellent though their achievements might be; and the cry of their chairman to-day was still "Excelsior." This feeling was the very part and parcel of hospital existence; they could not possibly stop where they were; the demands to be met, the evils to be grappled with, were evils which were increasing with the population. In conclusion, Sir William Hart-Dyke impressed on his hearers the fact that the wealthy people of the day needed stirring up and rousing to a sense of their responsibilities and to be told what is needed and what should be done. A vote of thanks was passed to the ladies who organised and did so much to ensure the success of the bazaar last year; and the Rev. C. J. Ridgway, in proposing a vote of thanks to the medical and surgical staff and to Mr. Empson, the honorary secretary, congratulated the committee on the useful work the hospital was doing. The total number of in-patients at the Paddington Green Hospital and the Convalescent Hospital at Harrow during 1895 was 298; and the total number of out-patient attendances, 39,661; and 2,602 casualty and accident cases.

### CROYDON BOROUGH HOSPITAL.

Up to 1891 the provision for infectious diseases at Croydon consisted of a temporary iron building situated near the sewage extractor containing twelve beds for small-pox, and an arrangement with the Board of Guardians for the treatment of scarlet fever cases in their infectious wards. The arrangement with the Guardians was terminated by the Local Government Board, and in 1891 the Corporation purchased eight acres of land situated in the angle formed by the western boundary of the borough and the Wimbledon Railway. The original iron buildings were transferred to this site, an administration block built, and subsequently two more iron buildings erected, raising the accommodation to 50 beds. These blocks have been in use since the spring of 1894. Since then additional brick buildings (providing for 50 fresh patients) have been erected, the wards have been connected by covered corridors, the roads have been constructed, and the site has been laid out. The hospital now consists of an administration block, diphtheria (22 beds), scarlet fever (acute) (22 beds), scarlet fever (convalescent) (24 beds and 2 cots), enteric fever and supplementary block (12 beds), block for paying patients (6 beds), probationary block, laundry, ambulance, disinfecting, and receiving rooms, mortuary, and bacteriological laboratory. Separated from the rest of the hospital, with a special building for the staff, is an emergency ward (12 beds), primarily intended for the reception of cholera. The new buildings were formally opened on the 6th inst. by the Chairman of the Sanitary Committee (Councillor King) and the Chairman of the Hospital Building Committee (Councillor Howard Martin). The two principal new blocks are similar in detail, and each consists of two light and airy wards, one containing eight beds for men and boys, and the other containing 12 beds for women and children. At the extreme end of each ward is a single-bedded ward for severe or delirious cases, and at the same end are the bath-room, w.c., lavatory, and other offices. The ward kitchen, the nurses' room, and the linen cupboard are situated between the two wards. The superficial area of floor space is 162 feet, and the cubic capacity 2,112 feet per bed. The wards are heated by means of open fires, there being a fire-place at each end and a stove in the middle of the ward. There is a ventilator under each bed for the admission of air, capable of being regulated by the nurses, and the foul air is extracted through ventilators at each side and end of the ward. The block for paying patients is made to accommodate six patients, and is so divided lengthways that different classes of disease can be perfectly separated from each other. Each side contains a kitchen in the centre, with a single-bedded room on one side and a room with two beds on the other, separate lavatory accommodation being provided for each room. The area per bed in these wards is 180 square feet and the cubical contents 2,340 feet. After a short opening ceremony, the visitors were shown over the new building by Dr. L. Wild, the Medical Officer of Health for Croydon, and the Matron, Miss Cootes.

